# Medical Leaders Identify Personal Characteristics and Experiences that Contribute to Leadership Success in Medicine

**DOI:** 10.15694/mep.2019.000206.1

**Published:** 2019-11-15

**Authors:** Louise Arnold, Paul Cuddy, Susan B Hathaway, Jennifer L Quaintance, Steven L Kanter

**Affiliations:** 1 The University of Missouri-Kansas City School of Medicine; 2 The Association of Academic Health Centers

**Keywords:** Medical leadership, characteristics of leaders, development of medical leaders, factors contributing to success throughout leaders’ careers

## Abstract

This article was migrated. The article was marked as recommended.

**Introduction:** The approach of medical educators to preparing learners for leadership reflects the emphasis leadership theories once placed on experiential learning. But, contemporary theories now also show a renewed interest in the role of personal characteristics in effective leadership. This shift raises questions explored here: What characteristics mark top medical leaders? What experiences nurture those characteristics?

**Method:** In a 2015 qualitative study, 48 University of Missouri-Kansas City (UMKC) medical graduates who met criteria for outstanding leadership participated in semi-structured interviews. Investigators applied directed content-analysis to their responses. Then, using iterative open-coding, investigators identified personal characteristics leaders said contributed to their leadership, clustered them into types, and counted the number of leaders who spoke to each type. Next, they coded and categorized experiences leaders discussed and counted the number of leaders who mentioned each type of experience. Finally, they identified leaders’ comments about which types of experiences helped develop which types of characteristics.

**Results:** Most leadersmentioned four types of characteristics: openness to new ideas/opportunities/astute risk-taking; intense motivation/active involvement/commitment; people-orientation; and capability/competence/ intelligence. Many discussed two additional types: self-awareness and service-orientation. Leaders said these types of experiences nurtured their characteristics: family traditions, high-school co-curricular activities, participation in medical school learning communities plus interaction with role models/mentors and authentic opportunities to practice leadership, innovation, and excellence throughout their education and in the workplace.

**Conclusions:** Medical leaders’ views of the role of personal characteristics in outstanding leadership and the power of educational and workplace experiences, especially informal ones, to mold those characteristics have enriched understanding how to prepare tomorrow’s leaders.

## Introduction

Complex changes in health care systems today underscore the need to develop physicians for leadership in medicine, a call the Institute of Medicine in the U.S.A. ( Institute of Medicine, 2004) and others ( Gunderman and Kanter, 2009) issued over a decade ago. The positive association between physician leadership and health care institutional performance furthers that need ( Goodall, 2011 and Postel *et al.*,2014). As a result, medical schools and professional organizations have accepted the challenge by offering leadership education in a variety of formal and less formal formats ( Arnold *et al*., 2018). Our recent study demonstrated the power of informal medical school experiences in preparing students for successful medical leadership ( Arnold *et al*., 2018).

The approach to leadership education found in schools and professional organizations matches leadership theories that once stressed the role of experiential learning in grooming tomorrow’s leaders ( McKimm and O’Sullivan, 2016 and Brown, 2013). However, contemporary theories now also show a renewed interest in the contribution of personal characteristics to effective leadership ( Brown, 2013) which in turn poses questions we explored from the perspective of medical leaders themselves: 1. What characteristics mark effective medical leaders? and 2. What experiences nurture those characteristics, particularly what types of experiences promote what types of personal characteristics?

## Methods

We took a phenomenological, qualitative approach to our study based on semi-structured interviews. These interviews yielded data to which we first applied directed content analysis. Because we already published detailed information about our study methods ( Arnold *et al*., 2018), we summarize below the main features of our methodology. Our university’s Institutional Review Board found the study to be exempt from further review and approval.

### Setting

The University of Missouri-Kansas City School of Medicine (UMKC) was the site for this research. Based on an innovative academic plan ( Drees, Arnold and Jonas, 2007), the school admits most of its students upon high school graduation into a six-year combined baccalaureate-MD curriculum ( Arnold, Ellison and Drees, 2010). From entry onward, students in small groups interact with patients, their families, physicians, and other health care providers. In their third year, students join a learning community known as a docent team, the focal point for learning and teaching how to think, feel, and act as a physician. The team cares for patients during a weekly continuing ambulatory care clinic and during an annual two to three-month inpatient internal medicine rotation.

Members of the docent team are: the team leader (a docent who is a general internist), other health care professionals, and three students from each of the third through the sixth year of the curriculum. Senior students form formal partnerships with junior team members. Students on the team, especially the partners, are expected to promote each other’s professional and personal development. They remain in the same partnership and on the same team until they graduate.

### Study sample

Study participants were among the 1,664 baccalaureate-MD graduates from the school’s first graduating class of 1976 through the class of 1999, the latest class with enough time to attain major leadership positions in medicine by the time we began the study (2015). To choose participants, we used two criteria for documented leadership achievement: 1. type of leadership role such as top administrator, outstanding clinician, distinguished researcher, and/or excellent educator and 2. type of institutional affiliation, such as national or regional professional associations/organizations, medical schools known for medical research and outstanding education, health care institutions recognized for excellent patient care, large government agencies involved with health care and medical research, large industrial companies related to health, etc. ( Arnold *et al*., 2018).

We identified 213 out of the 1,664 graduates as meeting our two leadership criteria ( Arnold *et al*., 2018). We invited 71 (33%) for interviews by prioritizing national-level achievement in a range of leadership roles in a variety of institutions ( Arnold *et al*., 2018). In August-November, 2015, we contacted these 71 leaders by e-mail or phone for interview; and 48 of them (68%) participated in interviews, primarily by phone ( Arnold *et al*., 2018).

### Data collection

We used a semi-structured interview guide to collect the data. It began with this open-ended question: “In your opinion, what has enabled you to become the leader in medicine that you are?” followed by these prompts: “Experiences before, during, and after medical school? People? Personal characteristics?” Interviewers took notes of the leaders’ responses. After finishing an interview, they transferred the leaders’ replies into an electronic document without any identifying information that could link a leader to his/her answers. The resultant document was ready for coding and entry into NVivo version 11 (QSR International, Burlington, Massachusetts) for analysis.

### Data analysis

We first conducted a directed content-analysis of the leaders’ responses to discover if the leaders implicated any personal characteristics in their leadership success. We conceptually defined personal characteristics as typical behavioral dispositions and habits, internal to the leader but not necessarily innate, that from the leader’s perspective describe the essence of who she or he is and that enable her/him to meet leadership needs of their organizations effectively. Using iterative open coding with careful attention to the reliability and validity of the process previously explained ( Arnold *et al*., 2018), we identified multiple personal characteristics that leaders said contributed to their leadership. Then, one author (L.A.) independently categorized the personal characteristics that seemed to cluster into types. Two authors (S.B.H. and J.L.Q.) independently verified the categorization. We resolved differences through discussion. Then, we counted the number of leaders who mentioned at least one characteristic within each type and converted the frequency counts into percentages.

Similarly, we used directed content-analysis to determine experiences that leaders considered influential in their leadership development. We conceptualized experiences to be comprised of events in which leaders were involved; their relationships with people, especially interactions with individuals the leaders considered highly influential; and their participation in organizational cultures. Again, using iterative open coding ( Arnold *et al*., 2018), we coded and categorized experiences the leaders discussed as impactful in their leadership development before, during, and after medical school. We counted the number of leaders who mentioned each type of experience and again transformed the count into a percentage. For this paper, we primarily focused on the experiences before and after medical school enrollment because our prior publication presented a detailed analysis of experiences during medical school ( Arnold *et al*., 2018). However, we will report in the results below two impactful medical school experiences, one already published ( Arnold *et al*., 2018), to gain a comprehensive picture of the types of experiences that influenced the development of the leaders’ characteristics.

Finally, we searched the interviews for the connections leaders saw, if any, between their experiences and the development of the personal characteristics that they thought contributed to their leadership. In particular, we noted their comments about the potential that each type of experience had for nurturing each type of characteristic.

## Results/Analysis

To present results, we followed conventions used in our previous article ( Arnold *et al*., 2018). That is, we converted percentages into descriptive quantitative statements because we believe percentages would attribute inappropriate precision to the results. Thus, when 80% of more of the leaders mentioned a characteristic or an experience, we report that “most leaders” did so. When 60% to 79% mentioned a characteristic or an experience, we report that “many leaders” did so. When around 50% of the leaders mentioned a characteristic or experience, we report that “about half” did so. When around 40% mentioned a characteristic or experience, we report that “some leaders” did so. When around 20% mentioned a characteristic or an experience, we report that “relatively few” did so; and when 10% or less of the leaders mentioned a characteristic or experience, we report that “few” leaders did so.

### Study participants

About half of the 48 interview participants were women. Most of them were in national-level leadership positions. Most were administrators. But, in addition to their leadership in administration, many also held leadership roles in research, patient care, and/or education. Most were affiliated with multiple types of organizations ranging from medical schools and universities, specialty societies and disease-focused organizations, through health care facilities/systems, to the military, government, and private practice. Details about the participants appear in a previous publication ( Arnold *et al*., 2018).

### Personal characteristics

We found six types of personal characteristics that the leaders said enabled them to become leaders in medicine.

**Type 1. Openness to new ideas and new opportunities and astute risk-taking.** Most of the leaders said that characteristics related to Type 1 were important for their leadership. Such characteristics as taking advantage of new opportunities, seizing the moment, and trying new things; being curious; being innovative, different, and non-conformist; thriving on challenges and risk-taking; changing career directions; continually learning; and being adaptable comprised this type.

**Type 2**. **Intense motivation, active involvement, commitment, and passion.** Most leaders also attributed their leadership success to characteristics connected with Type 2. Specific characteristics that constituted this type were being active and involved; being motivated and striving for excellence; working hard; being competitive, driven; being enthusiastic, passionate, and committed; having resilience, especially in the face of challenges; and being disciplined.

**Type 3**. **People-orientation.** In addition, most leaders mentioned characteristics associated with Type 3 as contributing to their leadership. Such characteristics as being skilled in communicating with others, listening, and building relationships; being collegial; having integrity; being humanistic, humble, fair, and loyal; leading by example; and surrounding oneself with supportive and enthusiastic people were part of people-orientation.

**Type 4. Capability, competence, preparedness, and intelligence.** Most leaders also pointed to Type 4 characteristics as influencing their leadership success. Specific characteristics included in this type were possessing decision-making ability; demonstrating competence; being well-prepared to assure competence; being smart/capable; being organized; being able to think clearly and critically; and having intellectual interests.

**Type 5**. **Self-awareness.** Many leaders thought characteristics associated with Type 5 played a role in their leadership. Being confident, knowing one’s self, being self-aware, being able to figure things out for oneself, and having high internal expectations made up the fifth type.

**Type 6**. **Service-orientation.** Finally, many leaders talked about the importance for their leadership of Type 6 characteristics comprised of these specific characteristics: wanting to help, to fix things, to be of service; promoting other people and guiding/establishing the next generation; having the ability to be a servant leader; and being a change agent.

Representative excerpts from the interview notes that illustrate these types appear in Table 1 below.

**Table 1.** Personal Characteristics Medical Leaders Said Contributed to Their Success

Six Types of Personal Characteristics	Specific Personal Characteristics Leaders Mentioned That Investigators Categorized into Type of Characteristic	Excerpts from Interviews
Type 1. Openness to New Ideas and New Opportunities, Astute Risk-taking	Seizing the moment, taking advantage of new opportunities, trying new things; Being curious; Being innovative, different, non-conforming; Thriving on challenges, risk-taking; Changing career direction; Continually learning, Being adaptable	20. *Personal characteristics contributing to your leadership? A pioneering spirit, adventuresome, willingness to take advantage of all the opportunities made available, open...*
Type 2. Intense Motivation, Active Involvement, Commitment, Passion	Being motivated, striving for excellence; Being active, involved; Driven and being competitive; Being a hard worker; Being persistent; Being committed, enthusiastic, passionate; Being disciplined; Being resilient	04. *[I] am highly motivated and active, those are characteristics looked for in the screening process; people who go to our school are like that; [I] don’t sleep a lot, don’t give up, am persistent. An example: tried to contact a patient multiple times, worked very hard at keeping the patient alive.*
		07. *Self-starter, driven to success, hard worker.*
Type 3. People-Orientation	Having good communication skills; Building relationships; Listening to others; Being humanistic; Collegial; Having integrity; Being transparent; Surrounding self with supportive people; Leading by example; Being humble; Being fair; Being loyal	12. *I am more touchy feely, focused on relationships. For me leadership is building teams, building relationships, not like in [my previous academic department] where the leadership is all top-down, the leader asks the questions, and the person under them does the work...*
Type 4. Capability, Competence, Intelligence, Preparedness	Being competent; Being able to make good decisions; Intellectual; Being well prepared; Being smart, not overly smart but having other good qualities; Being a clear or critical thinker; Being organized	15. *I felt very prepared for residency, and it was a tough residency as far as the complexity and volume of patients are concerned. Because I was so well prepared, I was noticed. And I was given all sorts of opportunities for leadership in residency...*
		13. *Being good at project management, excellent organizational skills, good coping mechanisms, figuring things out for yourself.*
Type 5. Self-Awareness	Having confidence; Having high internal expectations; Being able to figure things out for self; Having self-awareness, knowing self, being willing to act on self knowledge.	29. *Being interested in and believing I could do something [to serve]. The interview selected for certain qualities like a strong belief that you could do this, you could help people even though you were young, sure that you would spend your life making a contribution.*
Type 6. Service-Orientation	Wanting to help, fix things; Being service-oriented; Promoting others, establishing and guiding the next generation; Having the ability to be a servant leader; Being a change agent.	31. *Personal characteristics that contributed to [my] leadership ability? [Being] a servant leader.... The school looked for the best and brightest, but it looked for students who also are humanitarian, who will help each other out at three in the morning, who practice servant leadership.*

From interviews in 2015 with 48 University of Missouri-Kansas City School of Medicine Baccalaureate-MD graduates (1976-1999) who attained major leadership positions in medicine.

### Experiences

Leaders identified experiences they had before, during, and after medical school as influential in their leadership.

**Before medical school**. Most leaders thought that experiences they had before they came to medical school set a foundation for their leadership development, although a relatively few did not discuss pre-medical school experiences. Among the latter, they explained that it was difficult for them to remember or articulate how their early experiences might have contributed to their leadership.

But among those who did talk about experiences before they came to medical school, many pointed to the critical role their family played. Their parents in particular presented them with various family traditions such as community service, leadership, and involvement in education that influenced their leadership development. Parents, other family members, and family friends were role models and early mentors for leadership. This interview excerpt describes how parents provided support.

06. [Had] supportive parents who encouraged education, gave freedom to their children to choose [their own] path. Upbringing fostered independence.

Many leaders thought various high school experiences were important for their subsequent leadership. Many discussed various co-curricular activities as influential. Participation in sports, musical groups, science fairs, community projects, and student government, for example, or exposure to medicine through summer jobs in health care delivery or volunteer programs were some activities that the leaders described as offering them lessons about leadership. For some, but by no means all, their co-curricular activities gave them the initial opportunity to be in positions of leadership and to practice leading.

Experiences during high school also provided access to people beyond their family who were role models and mentors for leadership. High school teachers, coaches, heads of youth organizations, and community physicians served in this capacity.

**During medical school.** Co-curricular activities during medical school also figured into the leaders’ success. Some leaders talked about the influence of their participation in student organizations, especially university student government and governance bodies of the medical school, and their service as a leader in these activities. The following comment exemplifies the remarks of those leaders who were active in co-curricular affairs during the medical school.

36. Opportunities for students in organizations and school governance, committees, fraternities, search committees are important. Being a representative to the medical school student council, then its vice president, and being a student representative on the admissions committee [with voting privileges] enabled me to become a leader [among other influences].

These experiences gave our leaders opportunities to assume leadership positions as medical students and to exercise leadership skills.

We will not report here the many other ways, already published, that the leaders’ experiences in medical school contributed to their leadership development ( Arnold *et al*., 2018). However, we do include here the importance the leaders attributed to their membership as students in learning communities called docent teams. The leaders thought that was a critical factor in their subsequent success ( Arnold *et al*., 2018). For most, the team leader, a faculty member called a docent, was a role model of leadership and a mentor, sponsor, and source of inspiration for leadership ( Arnold *et al*., 2018). For many of the leaders, their junior-senior partnership between older and younger students on the docent team was another component of the learning community that enabled them to assume leadership positions subsequently ( Arnold *et al*., 2018). Longitudinal team experiences in clinical settings and in their assigned medical school team space were also formative ( Arnold *et al*., 2018). These interview excerpts convey how the docent team helped develop leaders.

28. The junior-senior partnership [was] most influential for leadership, [including] teaching others.

45. .. the docent team was a positive nurturing environment, [we had] progressive responsibility over the four years [for patient care, teaching others]. Senior partner couldn’t have been better.

48. You had an office, an identity, and peers [on the team]. You learned to work together - all very natural.. You had a senior partner and opportunity to teach your junior partner.

**After medical school, during residency/fellowship and in the workplace.** A pivotal experience occurred for many leaders early in residency ( Arnold *et al*., 2018). Their excellent medical school preparation for the supervised practice of medicine enabled them to stand out among their peers from the outset of their graduate medical education and to be noticed as potential leaders by faculty, residency directors, and peers. Consequently, the leaders received offers to assume leadership positions in their residency programs which they were willing and able to accept. In turn these early leadership positions became additional springboards to other leadership roles. This comment epitomizes the dynamic:

19. By the time we graduated we had more clinical exposure than graduates from other schools. That is what made us the best doctors. I mentioned that all that exposure gave us competence that affected our confidence that enabled us to stand out in the eyes of peers and attendings. They knew I had excellent bedside skills that made me an academic leader, and gave me leadership opportunities that set us up for more leadership. So clinical skills was key, seeing how good a doctor you can be.

It was not unusual for the leaders to have served as chief residents.

Indeed, many leaders spoke about the importance of holding authentic leadership positions in residency, fellowship, and the workplace as critical for their development. These positions, such as chief resident, afforded them the possibility to apply, practice, and hone their leadership skills.

According to many of the leaders, the environment of residency and fellowship programs as well as the environment of the institutions where the leaders worked helped to promote their leadership success. They singled out the importance of residency and fellowship environments that responded to their needs, motivations, and desires to pursue innovation, as well as environments that recognized their accomplishments, encouraged further achievement, and valued rigor and high standards.

To these environmental descriptors of supportive resident and fellowship environments they added the importance of these workplace features in advancing their leadership: an emphasis on excellence, collaborative relationships, and close involvement with talented colleagues and leaders in the field, along with opportunities to grow and build or create something new. All these environmental qualities heightened the leaders’ sense that they belonged in their program or workplace. Notably, leaders sought out a more nurturing environment in which to work when their workplace lacked these features.

Many leaders also mentioned the significance of role models and mentors in residency, fellowship, and the workplace for their leadership success. Mentors, especially, guided their leadership trajectory by offering formal and informal feedback, advising and counseling about personal and professional matters, and opening doors for them. Most leaders named specific peers, senior co-workers, teachers, advisors, supervisors, bosses, chiefs, department heads, and chairs as instrumental mentors.

Nearly half of the leaders talked about the importance of networks for leadership development. Some, after they completed their graduate medical education, mentioned the help they received from participation in formal leadership courses and in leadership programs their specialty societies held.

**Connections between experiences and personal characteristics.** As Tables 2 through 7 indicate below, experiences that leaders had before, during, and after medical school contributed to the development of each of the six types of characteristics that leaders believed enabled them to rise to top leadership positions. There were a very few exceptions to this generalization. A relatively few of the leaders could not speak to the role of early pre-medical school experiences, and a relatively few said that their experiences after medical school graduation were most influential.

**Table 2.** Types of Experiences Medical Leaders Said Contributed to Developing Type 1 Characteristics

Timing and Types of Experiences	Interview Excerpts Linking Types of Experiences and Characteristics
**Before Medical School**	
In the family: Family traditions; Family support; Family role models.	18. Moving around [as a child] helped me to see there *wasn’t just one way of doing things & furthered the idea of “thinking out of the box”* that my mother sparked with her interest in bringing about change & learning from the disadvantaged.
During high school: Participation in co-curricular activities; Opportunities to formally/informally lead; Teachers/coaches as role models & mentors.	10. I had a high school chemistry teacherwho showed me how to approach weighing opportunities, how to choose initial paths. He taught me that *things change and you have to take opportunities that come your way, perhaps even unexpectedly, and to have an open mind* * so you can seize those opportunities (while not taking every one just because they’re there)*.
**During Medical School** Participation in co-curricular activities; Opportunities to practice leadership skills; Membership on a health care team. ^b^	25. ... the entire staff was committed to making a part of our experience a sense of adventure. After all, the school was new. There was a sense of freshness. The environment was dramatically optimistic, energetic that led us to the idea that *we were pioneers, we were on an adventure,* we lacked fear.
**After Medical School**	
In residency/fellowship: Holding authentic leadership positions; Role models, mentors, sponsors; Programs supporting resident/fellow’s interests, etc. Environments stressing excellence, innovation.	39. Residency allowed him to do *what he was interested in - ethics*....They allowed residents to do interesting things... *things at the margin* .
In the workplace: Honing leadership skills in real leadership positions; Environments responsive to one’s goals, motivations; Environments stressing excellence, collaboration; Close involvement with talented colleagues/leaders; Role models, mentors; Access to networks.	24. The physician who recruited me to my current school was the ultimate role model and mentor....He opened doors for me. I was just in the right place at the right time with the right people. The school’s administration was supportive too; *it would let you go out on a limb and try things out of the ordinary*; it didn’t say ‘you can’t

From interviews in 2015 with 48 University of Missouri-Kansas City School of Medicine Baccalaureate-MD graduates (1976-1999) who attained major leadership positions in medicine. Type 1 characteristics: Openness to new ideas and new opportunities and astute risk-taking. ^a^

^a^ Experiences are underlined. Characteristics are italicized.

^b^ A supportive learning community called a docent team with role models & mentors.

**Table 3.** Types of Experiences that Medical Leaders Said Contributed to Developing Type 2 Characteristics

Timing and Types of Experiences	Interview Excerpts Linking Types of Experiences and Characteristics
**Before Medical School**	
In the family: Family traditions; Family support; Family role models & mentors.	17. My mother was a very big influence. She recognized my abilities and encouraged *me to get active in various ways*....She suggested that I should become active in activities outside of school. *And I did do several community projects*.
During high school: Participation in co-curricular activities; Opportunities to formally/informally lead; Teachers/coaches as role models & mentors.	03. [The leader] *reinvigorated the black student association in high school, was involved in speech and debate and other student activities....attempted to change school policies,* *3 ^rd^ year in high school changed student government,* had to get 10 names of students to support him to become a student senator, his classmates asked him to run for student senate, created special position for him within the student government...
**During Medical School** Participation in co-curricular activities; Opportunities to practice leadership skills; Membership on a health care team. ^b^	37. I was *always involved in something*....In medical school... was an ambassador [students chosen to represent the university, welcome visitors to Campus], in on-call singers [a medical student singing group], did research...throughout medical [with the same faculty researcher, on a non-for-credit basis].
**After Medical School**	
In residency/fellowship: Holding authentic leadership positions; Role models, mentors, sponsors; Programs supporting resident/fellow’s interests, etc.; Environments stressing excellence, innovation.	33. [The leader] *has been utterly committed to his clinical cause* that came about from his experiences in residency. He was appalled at what residents did not know and *that committed him to training others.*
In the workplace: Honing leadership skills in real leadership positions; Environments responsive to one’s goals, motivations; Environments stressing excellence, collaboration; Close involvement with talented colleagues/leaders; Role models, mentors; Access to networks.	40. Was recruited by a fellow graduate of the school to a major university with a medical school. Continually found himself surrounded by supportive people who welcomed *his enthusiasm* and helped to promote *his initiatives*.

From interviews in 2015 with 48 University of Missouri-Kansas City School of Medicine Baccalaureate-MD graduates (1976-1999) who attained major leadership positions in medicine. Type 2 characteristics: Intense motivation, active involvement, commitment, and passion. ^a^

^a^ Experiences are underlined. Characteristics are italicized.

^b^ A supportive learning community called a docent team with role models & mentors.

**Table 4.** Types of Experiences that Medical Leaders Said Contributed to Developing Type 3 Characteristics

Timing and Types of Experiences	Interview Excerpts Linking Types of Experiences and Characteristics
**Before Medical School**	
In the family: Family traditions; Family support; Family role models & mentors.	15. First off, my family had lots of strong leaders, not just my parents but aunts and uncles; seeing them be trail blazing leaders in different areas such as education, teaching, and diversity contributed to my interest in leadership and *watching them contributed to my [people] skills.*
During high school: Participation in co-curricular activities; Opportunities to formally/informally lead; Teachers/coaches as role models & mentors.	14. During the summers I worked for a small local suburban paper selling advertising. The owner of the paper was a friend of the family. As co-editor [of my high school paper] I *learned the importance of building relationships with the staff and how to do that.* And at the local paper as well as with the high school paper I *gained an understanding of the importance of communication and networking, all fundamental to leadership.*
**During Medical School** Participation in co-curricular activities; Opportunities to practice leadership skills; Membership on a health care team. ^b.^	27. We had a solid experience; it [the team] taught us how teams function effectively, how to relate, how to get along with even those you didn’t exactly love; *you knew that being a team member they would step up when needed and vice versa.* *It did that because the team stayed together for four years.* (Arnold *et al*., 2018)
**After Medical School**	
In residency/fellowship: Holding authentic leadership positions; Role models, mentors, sponsors; Programs supporting resident/fellow’s interests, etc.; Environments stressing excellence, innovation.	08. The program director of one of my ENT fellowships invested heavily in his residents. He did things for us that he didn’t have to do. He was fair, went to bat for you if you were abused in one of the other departments; he was very balanced in his treatment of us. *I vowed I would model that behavior if I had an opportunity to be a program director and I did train residents where I endeavored to pay it forward by the way I treated residents.*
In the workplace: Honing leadership skills in real leadership positions; Environments responsive to one’s goals, motivations; Environments stressing excellence, collaboration; Close involvement with talented colleagues/leaders; Role models, mentors; Access to networks.	18. My workplace has been very supportive of my work. It is a place that...fosters collaboration in cross-disciplinary work *so that I have been able to do my work on* *early childhood development [with faculty beyond my department].*

From interviews in 2015 with 48 University of Missouri-Kansas City School of Medicine Baccalaureate-MD graduates (1976-1999) who attained major leadership positions in medicine. Type 3 characteristics: A people-orientation. ^a^

^a^ Experiences are underlined. Characteristics are italicized.

^b^ A supportive learning community called a docent team with role models & mentors.

**Table 5.** Types of Experiences that Medical Leaders Said Contributed to Developing Type 4 Characteristics

Timing and Types of Experiences	Interview Excerpts Linking Types of Experiences and Characteristics
**Before Medical School**	
In the family Family traditions; Family support; Family role models & mentors.	24. My parents encouraged us, told us to *be the best that we could be* . There was the expectation *to succeed* *which I had*.
During high school Participation in co-curricular activities; Opportunities to formally/informally lead; Teachers/coaches as role models & mentors.	08. My experiences before enrollment groomed me to be a physician-leader. For much of my education before enrolling in the UMKC program I was in a very rigorous education leadership program. So *my academic preparation was excellent: 3 years of Latin, advanced math, etc. and when I came to Kansas City....I was exceedingly well prepared.*
**During Medical School** Participation in co-curricular activities; Opportunities to practice leadership skills; Membership on a health care team. ^b^	32. My year 1 docent was very formative for me as a clinician, for me maturing; he laid the foundation for clinical excellence -- the first step to becoming a leader in medicine. Then my Year 3-6 docent... created a clinical experience 2 ^nd^ to none that really prepared us as physicians for residency..... *In my first month of residency I was given the critical care unit, 3 patients to manage at once and I said I can do that because in medical school I had been given the autonomy to care for patients, with supervision of course.* Residents from other medical schools were lost. That made me a leader, gave me a definite advantage, set me up to be recognized as a leader & as a peer mentor, coach, & teacher that led to other leadership opportunities.
**After Medical School**	
In residency/fellowship Holding authentic leadership positions; Role models, mentors, sponsors; Programs supporting resident/fellow’s interests, etc; Environments stressing excellence, innovation.	28. I took a patient safety fellowship at a large well known hospital in that city. The CEO of that hospital and director of the fellowship spoke in such enthusiastic terms about their work in patient safety/quality that I decided I needed to work with them.... In that fellowship I learned *how to build teams, to understand people’s motives, to understand how fiscal things worked.... and to pick up skills for work in patient safety/quality.*
In the workplace Honing leadership skills in real leadership positions; Environments responsive to one’s goals, motivations; Environments stressing excellence, collaboration; Close involvement with talented colleagues/leaders; Role models, mentors; Access to networks.	26. The chair of the department where I was first a faculty member suggested that I had leadership potential. I took some courses, and then I got into a formal national program to groom women leaders. Then the dean’s office made me associate dean of faculty development which put me on everybody’s radar *and I had an opportunity to practice what I learned in that program.*

From interviews in 2015 with 48 University of Missouri-Kansas City School of Medicine Baccalaureate-MD graduates (1976-1999) who attained major leadership positions in medicine. Type 4 characteristics: Capability, competence, intelligence, and preparedness. ^a^

^a^ Experiences are underlined. Characteristics are italicized.

^b^ A supportive learning community called a docent team with role models & mentors.

**Table 6.** Types of Experiences that Medical Leaders Said Contributed to Developing Type 5 Characteristics

Timing and Types of Experiences	Interview Excerpts Linking Types of Experiences and Characteristics
**Before Medical School**	
In the family Family traditions; Family support; Family role models & mentors.	04. [ Her parents had emigrated to the US] Her mother taught her daughter that she had something to prove in this new country *, prove to other people [here] as well as yourself.*
During high school Participation in co-curricular activities; Opportunities to formally/informally lead; Teachers/coaches as role models, mentors.	21. My high school experience taught me as a woman that it was ok to compete and to be aggressive, and to be a leader *.* So I was being shaped *to take charge*....a personal characteristic [that contributed to success as a leader].
**During Medical School** Participation in co-curricular activities; Opportunities to practice leadership skills; Membership on a health care team. ^b^	43. Her docent was the first to give her feedback which conflicted with her self-image. The junior-senior partnership system was very valuable; especially *as a senior partner she came to understand why her own senior partner had trouble working with her.*
**After Medical School**	
In residency/fellowship Holding authentic leadership positions; Role models, mentors, sponsors; Programs supporting resident/fellow’s interests, etc; Environments stressing excellence, innovation.	23. I was just the third female to be board-certified in aerospace medicine. I was a flight surgeon in a fighter squadron. And faced with the attitude of ‘a woman can’t do this *’ I took the attitude of ‘yes I can,’ accepted the challenge, and was put into leadership positions. I had the attitude that I could do it, and I was willing to do it,* and was young enough to do it. I became chief resident.
In the workplace Honing leadership skills in real leadership positions; Environments responsive to one’s goals, motivations; Environments stressing excellence, collaboration; Close involvement with talented colleagues/leaders; Role models, mentors; Access to networks.	25. *I became and remain a self-starter*. By moving to my new chair position, I have an incredible opportunity of leaving a department 41 ^st^ in scholarly support to leading a department that is number 4 upon which to build even a better world-class program. I seized the opportunity.

From interviews in 2015 with 48 University of Missouri-Kansas City School of Medicine Baccalaureate-MD graduates (1976-1999) who attained major leadership positions in medicine. Type 5 characteristics: Self-awareness. ^a^

^a^ Experiences are underlined. Characteristics are italicized.

^b^ A supportive learning community called a docent team with role models & mentors.

**Table 7.** Types of Experiences that Medical Leaders Said Contributed to Developing Type 6 Characteristics

Timing and Types of Experiences	Excerpts from Interviews Linking Types of Experiences and Characteristics
**Before Medical School**	
In the family Family traditions; Family support; Family role models & mentors.	11. My family experience had a lot to do with leadership. I am the second oldest of six kids. My mom had the tradition of taking in people who needed a place to live; *that was just what you did*. [Also] My dad was killed in an accident and so my mother had to go to work. *My sister and I were largely responsible for our siblings.*
During high school Participation in co-curricular activities; Opportunities to formally/informally lead; Teachers/coaches as role models, mentors.	12. I went to a college prep Jesuit high school. Its motto was “men for others.” *So I had a junior and senior project which I did in a nursing home.* The message to the students was “ *you are here for others, to serve others*, and you will be leaders.”
**During Medical School** Participation in co-curricular activities; Opportunities to practice leadership skills; Membership on a health care team. ^b^	26. What ... impacted me were the opportunities I had to do volunteer work [in medical school] at the local children’s hospital, e.g. *I helped to raise money for a lot of causes to make things better*. It was fun and a release.
**After Medical School**	
In residency/fellowship Holding authentic leadership positions; Role models, mentors, sponsors; Programs supporting resident/fellow’s interests, etc.; Environments stressing excellence, innovation.	26. I *did that as a resident and I still do as a faculty member* [help raise money for causes to make things better.]
In the workplace Honing leadership skills in real leadership positions; Environments responsive to one’s goals, motivations; Environments stressing excellence, collaboration; Close involvement with talented colleagues/leaders; Role models, mentors; Access to networks.	16. We were in a city with a large military installation. Its health care facility was important. From being there I learned *humility* & *encountered servant leadership* everywhere one turned, how that was acted out, that it should inform everything you do.

From interviews in 2015 with 48 University of Missouri-Kansas City School of Medicine Baccalaureate-MD graduates (1976-1999) who attained major leadership positions in medicine. Type 6 characteristics: Service-orientation. ^a^

^a^ Experiences are underlined. Characteristics are italicized.

^b^ A supportive learning community called a docent team with role models & mentors.

Inspection of the tables further reveals regularities in the types of experiences that helped shaped the leaders’ personal characteristics before, during, and after medical school. These included the presence of supportive environments that inspired, recognized, and promoted the leaders’ motivations, abilities, high standards, and interests particularly in pursuing innovation and helping to make a difference. In addition, role models for leadership were readily available. Mentors, ranging from parents, teachers, docents, peers and colleagues, to supervisors and accomplished leaders in their disciplines, guided them, offered feedback and counsel, and connected them to leadership positions where they could practice and sharpen their leadership skills in authentic environments.

## Discussion

The types of characteristics leaders said contributed to their leadership success were: openness to new ideas, opportunities, and astute risk-taking; intense motivation, active involvement, commitment, and passion; a people-orientation; capability, competence, intelligence, and preparedness; self-awareness; and a service-orientation. By and large, these types of characteristics appear in the general and medical literature on characteristics of leaders, notably successful leaders. More specifically both the general and medical literature point to the role of openness, creativity, innovation, and forward-thinking as characteristics of effective leaders comparable to those we clustered in Type 1 ( Brown, 2013, Harolds, 2004 and Kouzes and Posner, 2012). Similarly, the literature recognizes commitment, drive and will power, determination, conscientious, and achievement orientation among characteristics of outstanding leaders that echo those in Type 2 ( Brown, 2013, Souba, 2011 and Vender, 2015). The importance of characteristics related to Type 3, a people-orientation, for success in leadership also appears in the literature. People-oriented characteristics identified in the literature include good interpersonal skills, listening, empathy, communication, social skills, cooperation, loyalty, integrity, plus a focus on building community and empowering people ( Brown, 2013, Harolds, 2004, Souba, 2011, Vender, 2015, Nonaka and Takeuchi, 2011 and Spears, 2010). The literature addresses characteristics related to those listed in Type 5: self-awareness including self-efficacy and self-confidence ( Brown, 2013, Souba, 2011 and Vender, 2015).

Although much of the literature parallels our findings about the characteristics top medical leaders believed were influential in their leadership, several nuanced differences emerged from a comparison of our results and reports in the literature. First, past articles have recognized as part of effective leadership variants of characteristics in Type 4 of capability, competence, intelligence, and preparedness such as talent, decision-making ability, and organization ( Brown, 2013, Kouzes and Posner, 2012 and Souba, 2011). However, we did not find in the literature the emphatic stress that most of our leaders put on the role of competence in their core discipline -- in this case, in medicine -- particularly early in their careers as a springboard to further leadership positions. Moreover, the leaders continued throughout their careers to pay attention to preparation to assure competence in their core discipline and in leadership. Second, the leaders added another dimension to openness to new ideas and new opportunities as conceptualized in the literature; they introduced the notion of being willing to take well-considered astute risks in pursuing new, pioneering adventures. Third, consideration of a service-orientation in leadership success primarily appears in the literature on servant leadership itself ( Spears, 2010 and Greenleaf (1970), whereas many of our medical leaders thought that their desire to help and be of service to others, to fix or change circumstances for the better -- in short to be a servant leader -- was fundamental to becoming a top leader. The importance they ascribed to service may well stem from the value that the medical profession places on service and the leaders’ embrace of that value as physicians. In all these ways, then, the ideas that medical leaders expressed in this study about characteristics associated with successful leadership have not only reinforced the leadership literature but also expanded it.

Further, the medical leaders have added to the literature in general and specific ways about how to prepare future leaders. In general, the experiences the leaders mentioned as impactful before, during, as well as after medical school exhibited similarities. Moreover, leaders implicated similar types of experiences at each phase of their lives in nurturing each of the types of characteristics. For example, before, during, and after medical school they found themselves in close relationships, groups, networks, and organizations that nurtured, supported, and advanced several characteristics. These were: their penchant for pursuit of innovation and new ideas; their motivations, interests, and passions; and their insistence on preparation, competence, and excellence. Also throughout, they had ready access to role models for leadership, to mentors, and to sponsors who guided their development and connected them to opportunities to lead that contributed to their people- and service-orientations.

Throughout, the preponderance of experiences leaders most often described were informal in nature, not part of formal training on leadership, but nonetheless offering them the possibility of learning about and exercising leadership. These experiences built the leaders’ confidence and motivation by satisfying basic psychological needs for competence, autonomy, and belonging, as expressed in self-determination theory ( Deci and Ryan, 2008), which in turn enabled them to excel early in residency, to be noticed, and to begin their journey toward outstanding leadership.

The perspectives of these leaders on experiences that helped them develop into successful medical leaders hold implications for medical educators. For medical school admissions committees, the remarks of the leaders recommend they attend to early experiences of applicants during their pre-college and college years. Experiences within the family of origin and during high school may provide clues to subsequent leadership.Exposure to broad-based liberal arts study in college should also be considered, as our previous article about our leaders ( Arnold *et al*., 2018) and another publication ( Machalec *et al*., 2018)have urged. For educators tasked with curricular design of medical schools, the experiences of these medical leaders endorse implementation of recent trends we detailed in our earlier article ( Arnold *et al*., 2018). These include clinical experiences early in the curriculum, longitudinal clinical experiences, and intentionally integrated clinical, basic, and behavioral sciences ( Arnold *et al*., 2018). The important role of longitudinal membership on docent teams in the success of the medical leaders supports the organization of the entire study body of a medical school into learning communities, such as societies or colleges, as a number of medical schools have done ( Smith *et al*., 2014).

Our results hold implications too for residency and fellowship directors along with administrators responsible for setting conditions in the workplacein health care facilities and academic medical centers. They would be well advised to ensure positive environments for learning and for performing the multiple duties demanded of employees.

Limitations to our study derive from undertaking this research in just one institution, at that, a U.S. medical school featuring a unique six-year curriculum combining liberal arts, basic science, and medicine in longitudinal clinically immersive learning communities. Our selection of participants emphasized formal leadership achievement; thereby effective informal leaders among graduates of this school may have been overlooked. Further, the study is retrospective and derived from leaders’ memories that may be faulty. Future research could address these deficits. For example, a larger, more inclusive set of graduates could be involved in testing the insights gleaned from this work. The design of a prospective study could counter reliance on leaders’ memories. Cross-cultural research could examine the validity of our findings about leadership characteristics and the experiences that nurture them.

## Conclusion

Qualitative study results from medical leader interviews significantly expand the literature on personal characteristics involved in outstanding leadership. Their insights on the power of educational and workplace experiences, including those that are informal, to mold those characteristics enrich our understanding of what it takes to prepare tomorrow’s leaders, particularly the future leaders in medicine.

## Take Home Messages

• Medical leaders thought these types of personal characteristics contributed to their leadership success: openness to new ideas/opportunities/astute risk-taking; intense motivation/active involvement/commitment; people-orientation; self-awareness; and service-orientation.
• These personal characteristics match those identified in the leadership literature. However, medical leaders placed greater stress on the importance of a service-orientation and on competence in a core discipline (in this instance, medicine) than does the general literature on leadership.
• Leaders implicated similar types of experiences before, during, and after medical school that nurtured, supported, and advanced each of the personal characteristic types.
• The preponderance of the experiences leaders most often described as influential were informal in nature, not part of formal training on leadership, but nonetheless offering them the possibility of learning about and exercising leadership.
• The perspectives of these leaders on experiences that helped them develop into successful medical leaders hold implications for medical educators on admissions and curriculum committees of medical schools, for directors of graduate medical education programs, and for administrators of health care facilities involved with the education of physicians.

## Notes On Contributors

**Louise Arnold, Ph.D.** is professor emerita and previous associate dean for medical education and research at the University of Missouri-Kansas City School of Medicine. Her research interests are medical professionalism, professional identity formation, program evaluation, and other aspects of undergraduate medical education.

**Paul Cuddy, Pharm.D.** is professor, Department of Internal Medicine, and vice dean at the University of Missouri-Kansas City School of Medicine. His research interests lie in undergraduate medical education, particularly instruction of medical students and medical students’ learning processes as well as topics in pharmacology.

**Susan B. Hathaway, Ph.D.** is associate professor, Department of Pediatrics, at the University of Missouri-Kansas City School of Medicine. Her research interests include leadership and graduate medical education.

**Jennifer L. Quaintance, Ph.D.** is associate professor, Department of Biomedical and Health Informatics, and assistant dean for assessment and quality improvement at The University of Missouri-Kansas City School of Medicine. Her research interests are assessment, program evaluation, professionalism, professional identity formation, and qualitative research methods.

**Dr. Steven L. Kanter, M.D.** is president and CEO of the Association of Academic Health Centers, Washington, D.C. Formerly, he was dean of The University of Missouri-Kansas City School of Medicine. His research interests in medical education stretch across the entire continuum from the pre-medical school phase through continuing education.

## References

[ref1] Arnold L. Ellison S. and Drees B.M. (2010) University of Missouri-Kansas City School of Medicine. Academic Medicine. 85(9 suppl), pp.S316–S320. 10.1097/ACM.0b013e3181e933a0 20736575

[ref2] Arnold L. Cuddy P.G. Hathaway S.B. Quaintance J.L. (2018) Medical school factors that prepare students to become leaders in medicine. Academic Medicine. 93(2), pp.274–282. 10.1097/ACM.0000000000001937 28991842

[ref3] Brown K.M. (2013) Trait Theory.in Irby B.J. Brown G. Lara-Alecio R. and Jackson S. (eds) The Handbook of Educational Theories. Charlotte, North Carolina: Information Age Publishing Incorporated, pp.897–901.

[ref4] Deci E.L. and Ryan R.M. (2008) Self-determination theory: A macro-theory of human motivation, development, and health. Canadian Psychology/Psychologie canadienne. 49(3), pp.182–185. 10.1037/a0012801

[ref5] Drees B.M. Arnold L. and Jonas H.S. (2007) The University of Missouri-Kansas City School of Medicine: Thirty-five years of experience with a nontraditional approach to medical education. Academic Medicine. 82(4), pp.361–369. 10.1097/ACM.0b013e3180332f33 17414192

[ref6] Goodall A.H. (2011) Physician-leaders and hospital performance: Is there an association? Social Science and Medicine. 73(4), pp.535–539. 10.1016/j.socscimed.2011.06.025 21802184

[ref7] Greenleaf R.K. (1970) The Servant as Leader. Indianapolis, Indiana: Robert K. Greenleaf Center.

[ref8] Gunderman R. and Kanter S.L. (2009) Perspective: Educating physicians to lead hospitals. Academic Medicine. 84(10), pp.1348–1351. 10.1097/ACM.0b013e3181b6eb42 19881420

[ref9] Harolds J. (2004) Selected important characteristics for enlightened medical leaders. Journal of the American College of Radiology. 1(5), pp.338–342. 10.1016/j.jacr.2003.12.035 17411598

[ref10] Institute of Medicine . (2004) Academic health centers: Leading change in the 21 ^st^ century. Academic Emergency Medicine. 11(7), pp.802–806. 10.1197/j.aem.2004.02.007 15231482

[ref11] Kouzes J.M. and Posner B.Z. (2012) The Leadership Challenge: How to Make Extraordinary Things Happen in Organizations. 5th edn. San Francisco, California: Jossey-Bass Incorporated.

[ref12] Machalec B. Cuddy M.M. Hafferty P. Hanson M.D. (2018) It’s happening sooner than you think: Spotlighting the pre-medical realm. Medical Education. 52(4), pp.359–361. 10.1111/medu.13531 29574960

[ref13] McKimm J. and O’Sullivan H. (2016) When I say .. Leadership. Medical Education. 50(9), pp.896–897. 10.1111/medu.13119 27562889

[ref14] Nonaka I. and Takeuchi H. (2011) The wise leader. Harvard Business Review. 89(5), pp.58–67,146. 21548419

[ref15] Postel M. Frank P.N. Barry T. Satou N. (2014) The cost of preventing readmissions: Why surgeons should lead the effort. The American Surgeon. 80(10), pp.1003–1006. 25264648

[ref16] Smith S. Shochet R. Keeley M. Fleming A. (2014) The growth of learning communities in undergraduate medical education. Academic Medicine. 89(6), pp.928–933. 10.1097/ACM.0000000000000239 24871245

[ref17] Souba W.W. (2011) The being of leadership. Philosophy, Ethics, and Humanities in Medicine. 6(5), pp.5–16. 10.1186/1747-5341-6-5 PMC3050817 21349187

[ref18] Spears L.C. (2010) Character and servant leadership: Ten characteristics of effective, caring leaders. The Journal of Virtues and Leadership. 1(1), pp.25–30. Available at: https://www.regent.edu/acad/global/publications/jvl/vol1_iss1/Spears_Final.pdf. ( Accessed: 5 November 2019).

[ref19] Vender R.J. (2015) Leadership: An overview. American Journal of Gastroenterology. 110(3), pp.362–367. 10.1038/ajg.2014.199 25047400

