# Supplementary material for: Medical Leaders Identify Personal Characteristics and Experiences that Contribute to Leadership Success in Medicine
Source: MedEdPublish (2016). 2019 Nov 15;8:206. [Version 1] doi: 10.15694/mep.2019.000206.1 (PMC10712527; doi:10.15694/mep.2019.000206.1)
Supplement: Peer review history

# Reviewer report: Reviewer response for version 1

**DOI:** 10.21956/mep.19883.r30077

**Published:** 2019-12-03

**Authors:** Barbara Jennings

**Affiliations:** 1 Norwich Medical School

This review has been migrated. The reviewer awarded 4 stars out of 5 I enjoyed reading this article and will recommend it to medical educationalists and students who are interested in the principles and practice of leadership. In an era of rapid change and massive expansion of health services, an understanding of the characteristics of successful leaders - and how we can support leadership is paramount.The article contains a very useful overview of recent literature and is clearly written and logically presented. There is so much rich data shared in the authors' thorough tabulation and analysis of results.One of the authors' aims was to consider the implications for future training - i.e. they consider how the findings might be applied. I think more circumspection is needed here given that the study focused on data from a cohort selected entirely for outstanding leadership traits (with no comparison group). I wondered if the limitations section of the discussion could have been expanded because of the real risk of selection/survivor bias with this approach - was this something that the authors considered?Other limitations are clearly articulated and I note the triangulation of the authors' conclusions with those from the wider literature.

Reviewer Expertise:

NA

No decision status is available

# Reviewer report: Reviewer response for version 1

**DOI:** 10.21956/mep.19883.r30079

**Published:** 2019-11-29

**Authors:** Tharin Phenwan

**Affiliations:** 1 University of Dundee

This review has been migrated. The reviewer awarded 5 stars out of 5 This topic is very interesting and I enjoy reading this a lot. The article is very easy to follow-through. The methodology section especially the analysis section was very detailed and easy to follow. Each table demonstrated each type of leadership well. The authors outlined the limitations of this work and suggestions for future works well, especially the informal leadership recognition.This article will be of interest to those who are interested in leadership training and curriculum planning.

Reviewer Expertise:

NA

No decision status is available

# Reviewer report: Reviewer response for version 1

**DOI:** 10.21956/mep.19883.r30076

**Published:** 2019-11-29

**Authors:** Balakrishnan Nair

**Affiliations:** 1 Centre for Medical Professional Development and University of Newcastle

This review has been migrated. The reviewer awarded 5 stars out of 5 I enjoyed reading this excellent research. If we have to improve our health care systems we need to create leaders . This training should start at undergraduate level and we have many take home messages from this study. I commend the buddy system they have introduced in undergraduate years. Medical education should be transformative .

Reviewer Expertise:

NA

No decision status is available

# Reviewer report: Reviewer response for version 1

**DOI:** 10.21956/mep.19883.r30078

**Published:** 2019-11-21

**Authors:** P Ravi Shankar

**Affiliations:** 1 American International Medical University

This review has been migrated. The reviewer awarded 5 stars out of 5 I enjoyed reading this well-written and informative article. Leadership is receiving a lot of attention and importance in the medical school curriculum. The methodology of the study has been well described. The authors’ findings point to experiences before, during and after medical school as important in developing leadership qualities. Early clinical exposure and the importance of learning teams have been stressed in the article. The docent team was an interesting concept bringing together faculty, other health professionals and students during different years of medical school. I suppose that the medical school and the teaching hospitals were within geographically adjacent campuses? For offshore Caribbean medical schools the issue of geographical separation of the basic science and clinical sites could be a challenge though technology does enable one to mitigate this problem to a certain extent. The importance of student associations and learning communities has been stressed throughout. Empowering students is important to create tomorrow’s leaders. The authors have identified the limitations of their study very well. The conclusions drawn seem to be supported by the study results. The study will be of special interest to medical educators and educational leaders at medical schools.

Reviewer Expertise:

NA

No decision status is available

# Reviewer report: Reviewer response for version 1

**DOI:** 10.21956/mep.19883.r30075

**Published:** 2019-11-18

**Authors:** Sateesh Babu Arja

**Affiliations:** 1 Avalon University School of Medicine

This review has been migrated. The reviewer awarded 5 stars out of 5 A very interesting piece of work. Even professional organizations like the CanMEDS framework for physicians defined leadership as one of the required competencies for physicians. I thoroughly enjoyed reading this manuscript. The literature review is up to date and relevant. The research methodology is appropriate. Data analysis and results are adequate and appropriate. The authors explained the personal characteristics attributed to these leaders. The authors also detailed the factors fostering the leadership qualities before medicals school, during medical school, and after medical school. This paper is beneficial for academic administrators to maintain the learning environments which can foster leadership qualities.

Reviewer Expertise:

NA

No decision status is available
